# Dysregulated Collagen Homeostasis by Matrix Stiffening and TGF-β1 in Fibroblasts from Idiopathic Pulmonary Fibrosis Patients: Role of FAK/Akt

**DOI:** 10.3390/ijms18112431

**Published:** 2017-11-16

**Authors:** Alícia Giménez, Paula Duch, Marta Puig, Marta Gabasa, Antoni Xaubet, Jordi Alcaraz

**Affiliations:** 1 Unit of Biophysics and Bioengineering, Department of Biomedicine, School of Medicine, Universitat de Barcelona, 08036 Barcelona, Spain; alicia.gimenez.bio@gmail.com (A.G.); pduchgili@gmail.com (P.D.); mpuigmartinez@gmail.com (M.P.); aeryn13@gmail.com (M.G.); 2 Institut d’Investigacions Biomèdiques August Pi i Sunyer (IDIBAPS), 08036 Barcelona, Spain; axaubetmir@gmail.com; 3 Pneumology Service, Hospital Clínic, 08036 Barcelona, Spain; 4 CIBER de Enfermedades Respiratorias (CIBERES), 28029 Madrid, Spain

**Keywords:** pulmonary fibrosis, collagen, MMP1, matrix rigidity, TGF-β, FAK, Akt, fibroblasts

## Abstract

Idiopathic pulmonary fibrosis (IPF) is an aggressive disease in which normal lung parenchyma is replaced by a stiff dysfunctional scar rich in activated fibroblasts and collagen-I. We examined how the mechanochemical pro-fibrotic microenvironment provided by matrix stiffening and TGF-β1 cooperates in the transcriptional control of collagen homeostasis in normal and fibrotic conditions. For this purpose we cultured fibroblasts from IPF patients or control donors on hydrogels with tunable elasticity, including 3D collagen-I gels and 2D polyacrylamide (PAA) gels. We found that TGF-β1 consistently increased *COL1A1* while decreasing *MMP1* mRNA levels in hydrogels exhibiting pre-fibrotic or fibrotic-like rigidities concomitantly with an enhanced activation of the FAK/Akt pathway, whereas FAK depletion was sufficient to abrogate these effects. We also demonstrate a synergy between matrix stiffening and TGF-β1 that was positive for *COL1A1* and negative for *MMP1*. Remarkably, the *COL1A1* expression upregulation elicited by TGF-β1 alone or synergistically with matrix stiffening were higher in IPF-fibroblasts compared to control fibroblasts in association with larger FAK and Akt activities in the former cells. These findings provide new insights on how matrix stiffening and TGF-β1 cooperate to elicit excessive collagen-I deposition in IPF, and support a major role of the FAK/Akt pathway in this cooperation.

## 1. Introduction

Idiopathic pulmonary fibrosis (IPF) is a very aggressive rare disease in which the normally soft lung parenchyma is progressively and irreversibly replaced by a stiff dysfunctional scar [1]. Although its etiology remains unknown, IPF has been associated with the appearance of fibroblast foci, which are areas rich in activated fibroblasts/myofibroblasts in the background of excessive collagen deposition (mostly type I collagen) indicative of active fibrosis [1,2]. Likewise the excessive accumulation and pathologic activation of fibroblasts are hallmarks of fibrotic processes in other organs [3]. Accordingly there is growing interest in understanding what drives myofibroblast misbehavior and their associated aberrant collagen deposition in IPF and other types of organ fibrosis.

Type I collagen (collagen-I) is the most abundant extracellular matrix (ECM) component in both normal and fibrotic conditions. Collagen-I has a triple helix structure that arises from two α-1 and one α-2 chains, which are the products of the *COL1A1* and *COL1A2* genes, respectively [4,5]. The accumulation of collagen-I is thought to arise from an imbalance between collagen expression by fibroblasts and its degradation through collagenases [6,7]. Among the latter, the matrix metalloproteinase 1 (MMP-1) is the archetype of secreted collagenase [8]. In addition collagen-I can be degraded by other secreted MMPs including MMP-2, MMP-8 and MMP-13 [9,10]. However MMP-1 and MMP-2 are the most highly expressed collagenolytic MMPs in IPF [1,8].

Although the mechanisms underlying the excessive collagen deposition in IPF remain poorly understood, such aberrant deposition has been associated with persistent fibroblast activation [11,12]. Transforming growth factor beta-1 (TGF-β1) is the most potent fibroblast activator known to date, and has been pointed as a key pro-fibrotic cytokine in organ fibrosis in general and IPF in particular [11,12,13]. Intriguingly, *in vitro* studies have revealed that exogenous TGF-β1 triggers a transcriptional program in normal fibroblasts that favors collagen deposition by increasing *COL1A1* while decreasing *MMP1* expression [14,15], although the underlying mechanisms remain poorly defined. In addition to TGF-β1, there is solid evidence that ECM stiffening can also act as a potent pro-fibrotic mechanical stimulus [16], and may enhance *COL1A1* expression in fibroblasts *per se* [7,17,18]. Moreover, it should be noted that previous in vitro studies on the inverse (transcriptional) regulation of *COL1A1* and *MMP1* by TGF-β1 were carried out in fibroblasts cultured on standard two-dimensional (2D) tissue culture plastic, which is an extremely rigid material with a characteristic stiffness that is several orders of magnitude higher than any normal or fibrotic tissue [19,20].

Since fibroblasts are very sensitive to their local mechanical microenvironment, it is conceivable that matrix rigidity modulates inverse (transcriptional) regulation of *COL1A1* and *MMP1* by TGF-β1 in fibroblasts. However the nature of the interaction between matrix rigidity and TGF-β1 and how it becomes altered in IPF remain poorly understood. To address this gap of knowledge, we cultured primary pulmonary fibroblasts from control tissues and IPF patients in hydrogels with tunable elasticity in the absence or presence of TGF-β1, and examined the expression of both *COL1A1* and two important collagenases that are upregulated in IPF (i.e., *MMP1* and *MMP2*). Moreover, we examined the potential roles of focal adhesion kinase (FAK) and Akt in matrix rigidity modulation of TGF-β1 regulation of collagen homeostasis in normal and IPF-fibroblasts.

## 2. Results

### 2.1. Optimizing 2D and 3D Hydrogels with Tunable Elasticity to Mimic Normal and Pro-Fibrotic Mechanical Microenvironments

**Figure 1:**
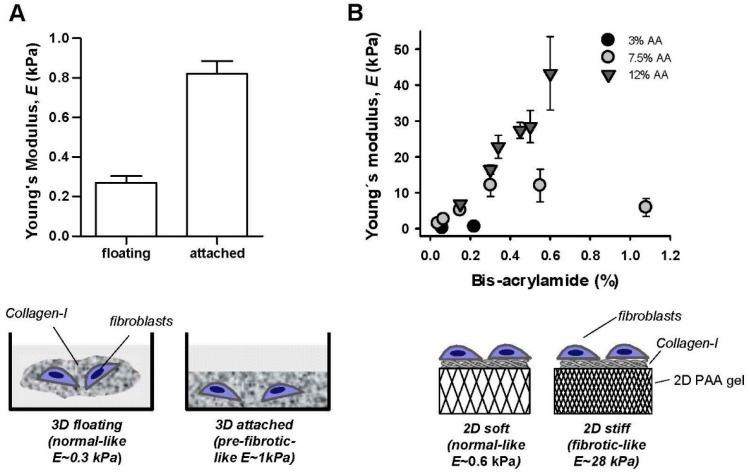
Cell culture substrata with tunable elasticity. (**A**) Top: Young’s elastic modulus of 3D floating/attached 4 mg/mL collagen-I gels assessed by AFM nanoindentation measurements. Bottom: outline of the floating/attached gel assay. (**B**) Top: Young’s elastic modulus of collagen-I coated 2D polyacrylamide (PAA) gels prepared using different acrylamide (AA) and bis-acrylamide concentrations probed by AFM nanoindentation measurements. Bottom: outline of the 2D PAA gel assay.

Mammalian pulmonary tissue is rather soft and elastic, with a stiffness defined by a Young’s elastic modulus (*E*)—which is the physical parameter commonly used to characterize a sample’s resistance to deformation [21]—in the range of ~3–10 kPa in bulk [22,23,24,25] and ~0.1–5 kPa at the nanometer scale [7] as summarized in Table 1. Normal tissue elasticity becomes compromised in pulmonary fibrosis, with a 2–3 fold increase in bulk tissue stiffness [24,26], and up to 30-fold stiffening at the nanometer-scale [7]. To culture fibroblasts in conditions that mimic the fibrotic mechanical microenvironment, we adapted two independent and complementary assays based on collagen-rich hydrogels with tunable elasticity as outlined in Figure 1. Both assays have been widely used in previous mechanobiology studies [27,28]. The floating gel assay is based on culturing fibroblasts embedded in three-dimensional (3D) collagen-I gels that remain either attached to their container or floating, thereby providing high or low mechanical resistance, respectively. Based on our previous work, we used dense collagen-I gels at the highest accessible concentration (4 mg/mL) to achieve the largest stiffness, which corresponds to *E* = 0.82 ± 0.19 kPa as assessed by nanoindentation measurements by atomic force microscopy (AFM) [29]. Likewise previous AFM measurements carried out by us and others have reported an average 3.3-fold higher *E* in attached compared to floating gel conditions [27,30,31]. Using the latter value we computed the expected *E* for floating 4 mg/mL collagen-I gels as shown in Figure 1A, which remained within the normal-like (floating) or pre-fibrotic-like (attached) range as summarized in Table 1, but failed to reach fibrotic values. Alternatively we prepared two-dimensional (2D) collagen-I coated polyacrylamide (PAA) gels at different acrylamide and bis-acrylamide concentrations. AFM measurements on PAA gels are shown in Figure 1B and revealed that these gels cover the physiopathologic range of *E* values [20,21]. Based on these data, we chose PAA gels exhibiting 0.6 and 28 kPa as models for normal-like and fibrotic-like rigidities, respectively, in subsequent experiments.

### 2.2. Combined Effect of TGF-β1 and Matrix Stiffening in the Expression of COL1A1 and Collagenolytic MMPs in Normal Fibroblasts Cultured in Dense 3D Collagen-I Gels

**Figure 2:**
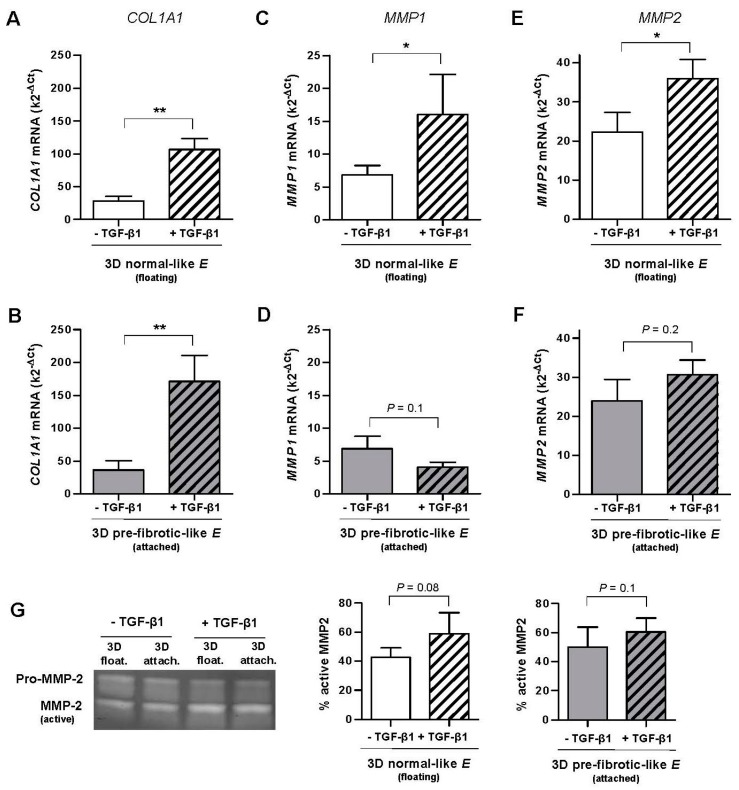
Effect of TGF-β1 on the mRNA levels assessed by qRT-PCR of *COL1A1* and MMP collagenases in primary pulmonary fibroblasts derived from control tissue (*n* = 3, patients with spontaneous pneumothorax) cultured in 3D floating/attached collagen-I gels. (**A**,**B**) *COL1A1* mRNA levels in floating (**A**) and attached (**B**) gels. (**C**,**D**) *MMP1* mRNA levels in floating (**C**) and attached (**D**) gels. (**E**,**F**) *MMP2* mRNA levels in floating (**C**) and attached (**D**) gels. (**G**) Representative gelatin zymogram showing the activity of secreted MMP2 (left panel). Corresponding quantification of the percentage of active MMP2 with respect to total MMP2 (pro-MMP2 + MMP2) (right panel). * *p* ≤ 0.05, ** *p* ≤ 0.01, *** *p* ≤ 0.005 here and thereafter were determined by paired Student’s *t*-test. Unless otherwise indicated, all fibroblasts were treated with TGF-β1 for ~4 days here and thereafter.

Exogenous TGF-β1 was added to 3D cultures and maintained for ~4 days, which is enough time to diffuse throughout the collagen-I hydrogels [34]. TGF-β1 enhanced substantially *COL1A1* expression in primary fibroblasts derived from control tissue (*n* = 3) cultured in 3D collagen-I gels, increasing the mRNA levels by ~300% in normal-like (floating) gels (Figure 2A) and by ~400% in pre-fibrotic-like (attached) gels (Figure 2B) with statistical significance. In addition we noticed that the total mRNA levels were moderately higher in attached compared to floating gels in either the absence (~25%) or presence (~50%) of TGF-β1, thereby suggesting a positive synergy between matrix stiffening and TGF-β1. These results reveal that the increase in *COL1A1* expression by TGF-β1, which has been extensively reported in standard 2D tissue culture plastic [7,17], can be extended to the soft normal-like microenvironment elicited by 3D collagen-I gels.

Unlike *COL1A1*, TGF-β1 induced a down-regulation of *MMP1* in pre-fibrotic (attached) gels (~−40%, Figure 2D), although it did not attain statistical significance. In contrast, TGF-β1 enhanced the average *MMP1* mRNA levels by ~140% in normal-like (floating) gels with statistical significance, and such increase was consistently observed in fibroblasts from all donors (*n* = 3) (Figure 2C). These findings reveal for the first time that the inverse (transcriptional) regulation of *COL1A1* and *MMP1* by TGF-β1 reported in stiff culture substrata here and elsewhere [14,15] does not hold in soft 3D collagen-I gels exhibiting normal-like stiffness.

Unlike *MMP1*, *MMP2* increased slightly upon TGF-β1 stimulation in floating (~60%) and attached (~30%) gels, although such increase attained only statistical significance in the former conditions (Figure 2E,F). Moreover, unlike *MMP1* and *COL1A1*, *MMP2* exhibited very similar mRNA levels in both floating and attached gels. Similar percentages of active MMP2 and a weak regulation by TGF-β1 in both floating and attached conditions were also observed by gelatin zymography (Figure 2G, right panels). Given the weak transcriptional modulation of *MMP2* by either TGF-β1 or matrix stiffening, we restricted our analysis to *COL1A1* and *MMP1* in subsequent experiments.

### 2.3. Combined Effect of TGF-β1 and Matrix Stiffening in the Expression of COL1A1 and MMP1 in Normal Fibroblasts Cultured in 2D Collagen-I Coated PAA Gels

**Figure 3:**
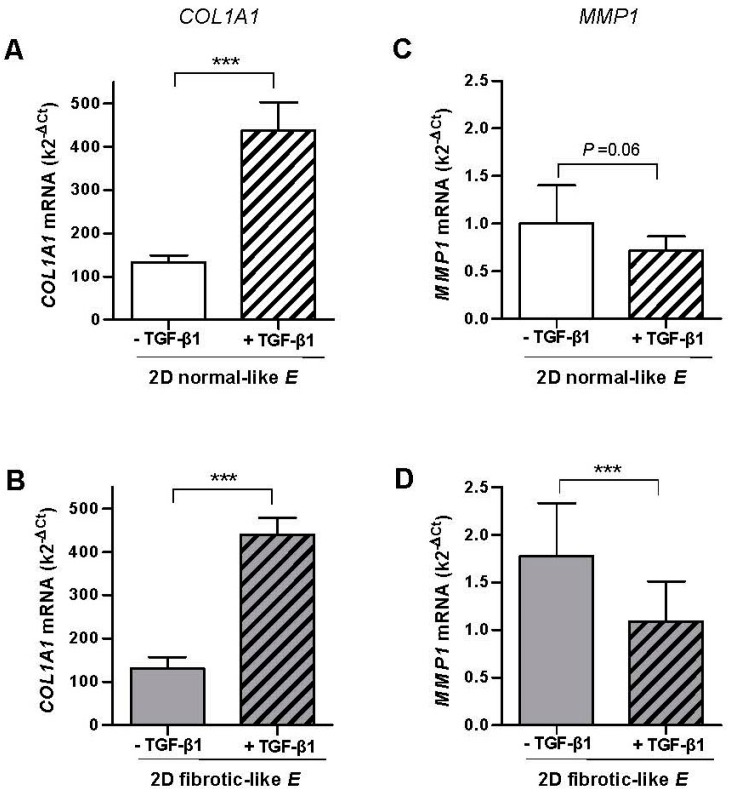
Effect of TGF-β1 on the mRNA levels assessed by qRT-PCR of *COL1A1* and MMP1 in CCD-19Lu fibroblasts and primary pulmonary fibroblasts derived from uninvolved regions of surgical lung cancer patients (*n* = 6) cultured in collagen-I coated 2D PAA gels. (**A**,**B**) *COL1A1* mRNA levels in gels exhibiting normal-like (**A**) or fibrotic-like (**B**) rigidities as shown in Figure 1B. (**C**,**D**) Corresponding *MMP1* mRNA levels of fibroblasts cultured as in B-C. Statistical analysis as in Figure 2.

To analyze the fibroblast transcriptional responses to TGF-β1 in a fibrotic-like mechanical microenvironment, fibroblasts from control tissue where cultured in 2D collagen-I coated PAA gels exhibiting either normal-like or fibrotic-like stiffness as described in Figure 1B. As in 3D cultures, TGF-β1 enhanced the mRNA expression of *COL1A1* in both soft (normal-like) and stiff (fibrotic-like) PAA gels with statistical significance (Figure 3A,B). However, unlike 3D cultures, both the increase in *COL1A1* mRNA induced by TGF-β1 (~230%) and the range of mRNA levels were very similar regardless the stiffness of the PAA gel. In addition we noticed that the mRNA levels were on average ~3.5-fold higher in 2D PAA gels compared to 3D collagen-I gels, revealing that the dimensionality of the microenvironment modulates the transcriptional activity of TGF-β1.

Unlike 3D cultures, TGF-β1 down-regulated *MMP1* mRNA in both 2D soft (normal-like) and stiff (fibrotic-like) PAA gels by ~−30% and~−40%, respectively, and such down-regulation was statistical significant in the latter conditions (Figure 3C,D). In addition the range of *MMP1* mRNA was slightly higher in stiff compared to soft PAA gels, and overall it was on average ~9-fold higher in 3D cultures compared to 2D PAA gels, further expanding that the transcriptional activity of TGF-β1 is modulated by the dimensionality of the culture conditions.

Altogether our results reveal that TGF-β1-dependent inverse regulation of *COL1A1* and *MMP1* mRNAs is similar qualitatively but not quantitatively in the stiff conditions elicited by either pre-fibrotic 3D collagen-I gels or fibrotic-like 2D PAA gels. In contrast the mRNA levels of *MMP1* induced by TGF-β1 in the softest conditions were strongly dependent on the dimensionality of the culture assay.

### 2.4. Linear Modeling of the Combined Effect of TGF-β1 and Matrix Stiffening in Gene Expression

To analyze quantitatively the potential interaction between TGF-β1 and matrix stiffening in regulating the mRNA of a specific gene (*R*), we used a simple linear model in the form:

$$R=R_{o}+a\Delta E+b\Delta C+\xi \Delta E\Delta C$$

where *R_o_* is the basal mRNA expression in a soft microenvironment in the absence of TGF-β1, Δ*E* and Δ*C* are the increase in either gel rigidity (*E*) or TGF-β1 concentration (*C*), *a* and *b* are proportionality factors between gene expression and either stiffness (*a*) or TGF-β1 concentration (*b*), and *ξ* is the interaction or coupling factor between stiffness and TGF-β1 concentration. The relationship between the experimental values of each condition and the model is as follows: *R_soft_* = *R_o_*, *R_soft+TGF-β1_* = *R_o_ + b*Δ*C*, *R_stiff_* = *R_o_ + a*Δ*E* and *R_stiff+TGF-β1_* = *R_o_ + a*Δ*E + b*Δ*C + ξ*Δ*E*Δ*C*.

According to our linear model, the ratio between the difference of gene expression with or without TGF-β1 in soft (Δ*R_soft_* = *R_soft+TGF-β1_* − *R_soft_*) and stiff (Δ*R_stiff_* = *R_stiff+TGF-β1_* − *R_stiff_*) gels should be

$$\frac{\Delta R_{stiff}}{\Delta R_{soft}}=1+\frac{\xi }{b}\Delta E\neq 1$$

Therefore, the prediction of the model is that Δ*R_stiff_*/Δ*R_soft_ =* 1 in the absence of interaction (i.e., *ξ* = 0), whereas Δ*R_stiff_*/Δ*R_soft_* ≠ 1 otherwise (*ξ* ≠ 0).

**Figure 4:**
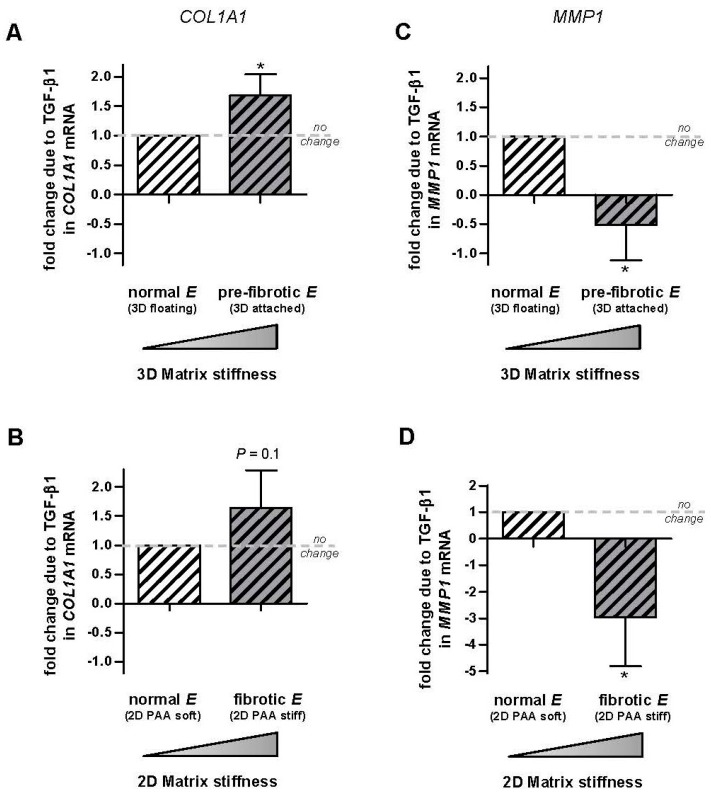
Analysis of the potential transcriptional synergy between TGF-β1 and matrix stiffening on *COL1A1* and *MMP1* in control primary pulmonary fibroblasts. (**A**,**B**) Average difference in *COL1A1* expression with our without TGF-β1 measured in soft and stiff substrata in each patient and normalized to the corresponding difference obtained in soft substrata using 3D collagen-I gels (**A**) or 2D PAA gels (**B**). Patients were the same than those used in Figure 2 and Figure 3. (**C**,**D**) Corresponding fold change in *MMP1*. Horizontal dashed line indicates no fold change. Further details are given in the main text. * *p* ≤ 0.05 with respect to no change (1) were determined by Student’s *t*-test.

To compare the predictions of the model with our data, we computed the ratio Δ*R_stiff_*/Δ*R_soft_* for each patient and assay, and the corresponding averages are shown in Figure 4. These computations revealed that the average ratio Δ*R_stiff_*/Δ*R_soft_* was consistently ~1.5-fold higher for *COL1A1* in the stiffest conditions elicited by both 3D cultures (Figure 4A) and 2D PAA gels (Figure 4B), although it attained statistical significance in the former assay only. Likewise, Δ*R_stiff_*/Δ*R_soft_* for *MMP1* was consistently <−0.5 lower and statistically significant in the stiffest conditions in both assays (Figure 4C,D). Therefore this analysis demonstrates that TGF-β1 and matrix stiffening do synergize at the transcriptional level positively for *COL1A1* and negatively for *MMP1*. In contrast we found only a modest positive synergy between TGF-β1 and matrix stiffening for *MMP2* in 3D cultures (Supplementary Materials Figure S1).

### 2.5. Inverse Regulation of COL1A1 and MMP1 mRNA Levels by TGF-β1 Is Associated with the Activation of the FAK/Akt Pathway

**Figure 5:**
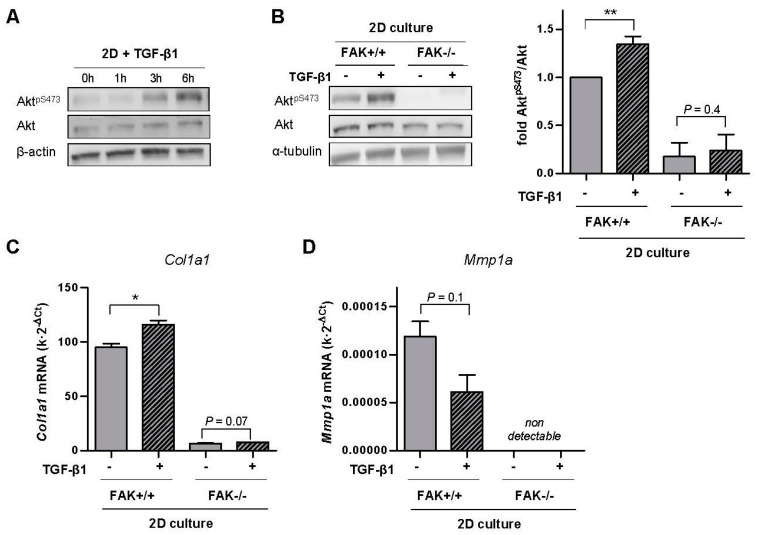
Analysis of FAK-Akt crosstalk in the inverse transcriptional regulation of *Col1a1* and *Mmp1a* by TGF-β1 in stiff microenvironments in FAK wild-type (FAK^+/+^) and FAK null (FAK^−/−^) fibroblasts. (**A**) Representative Western blot showing the time-course expression of Akt^pS473^, total Akt and β-actin in fibroblasts stimulated with TGF-β1 up to 6 h. (**B**) Representative Western blot showing Akt^pS473^, total Akt and α-tubulin of FAK^+/+^ and FAK^−/−^ fibroblasts stimulated with TGF-β1 for 6 h; (right) corresponding densitometry analysis of Akt^pS473^/Akt (*n* = 3). (**C**,**D**) Corresponding mRNA levels of *Col1a1* (**C**) and *Mmp1a* (**D**) of fibroblasts stimulated with TGF-β1 for ~4 days. *Mmp1a* could not be detected in FAK^−/−^ fibroblasts. Two-group comparisons were performed by Student’s *t*-test.

A large body of work has underlined the key role of integrin ECM receptors in sensing the mechanical microenvironment and transducing it into biological responses in fibroblasts and other cell types [27,35,36]. Moreover, we recently reported that matrix stiffening alone increases integrin mechanosensing in lung fibroblasts through phosphorylation of focal adhesion kinase (FAK) at tyr397 (FAK^Y397^), which in turn enhanced the activity of its downstream target Akt [37] through phosphorylation at S473 (Akt^pS473^) [35]. In addition, other groups have shown that TGF-β1 increases both FAK^Y397^ and Akt^pS473^ in fibroblasts in 2D cultures [38]. Moreover, Akt inhibitors have consistently elicited a reduction in *COL1A1* concomitantly to an increase in *MMP1* in fibroblasts [15]. Therefore all these previous observations raise the possibility that the inverse regulation of *COL1A1* and *MMP1* by TGF-β1 may involve the FAK/Akt pathway. To examine this possibility we first conducted a time-course analysis of Akt activation by phosphorylation at Ser473 upon TGF-β1 stimulation in fibroblasts cultured in standard tissue culture plastic substrata (i.e., 2D culture), and found a robust Akt activation at 6 h (Figure 5A and Supplementary Materials Figure S2). Next we took advantage of a well-established genetic model based on mouse embryonic fibroblasts that are either FAK wild-type (FAK^+/+^) or FAK null (FAK^−/−^) [39]. TGF-β1 stimulation induced an increase in Akt^pS473^ after 6 h in FAK^+/+^ but not in FAK^−/−^ fibroblasts cultured in the stiff conditions provided by standard 2D cultures (Figure 5B). Likewise 4-day treatment with TGF-β1 increased *Col1a1* while decreasing *Mmp1a* mRNA levels in FAK^+/+^ fibroblasts (Figure 5C,D), which are the mouse equivalent to *COL1A1* and *MMP1*, respectively. In contrast mRNA levels of *Col1a1* where two orders of magnitude lower in FAK^−/−^ compared to FAK^+/+^ fibroblasts and failed to increase upon TGF-β1 stimulation (Figure 5C), whereas *Mmp1a* could not be detected in FAK^−/−^ fibroblasts even after increasing the amount of cDNA by 5-fold on the qRT-PCR system (Figure 5D). These results reveal that FAK is necessary for the inverse regulation of *Col1a1* and *Mmp1a* mRNA levels by TGF-β1 in stiff microenvironments, and is associated with increased Akt activity.

### 2.6. Fibroblasts from IPF Patients Exhibit Aberrant Transcriptional Responses to TGF-β1 and Matrix Stiffening in Terms of Both COL1A1 and MMP1 Concomitantly with Enhanced Activation of the FAK-Akt Pathway

**Figure 6:**
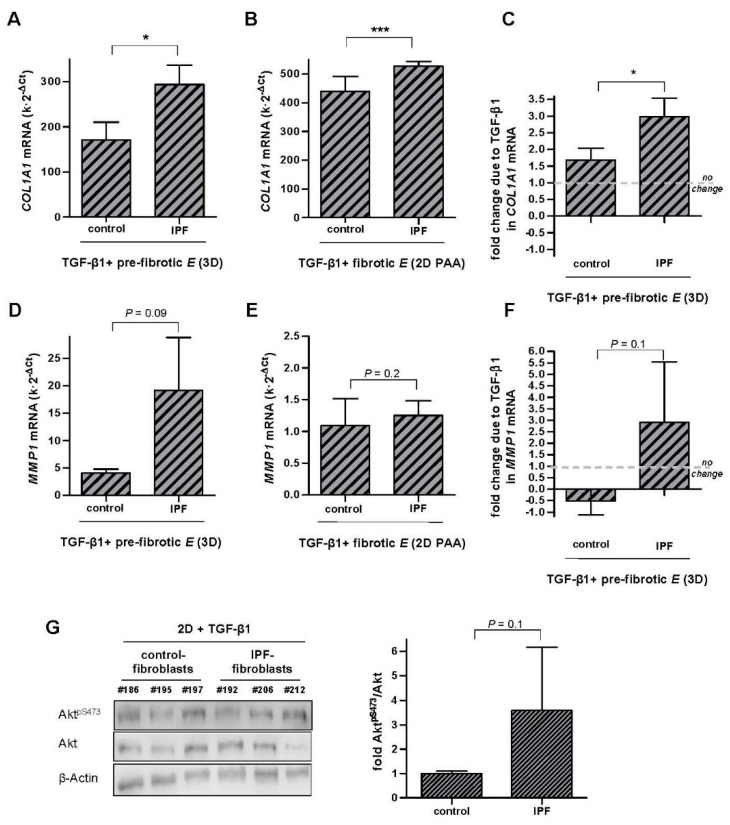
Aberrant transcriptional regulation of *COL1A1* and *MMP1* elicited by pro-fibrotic conditions (TGF-β1 and stiff substrata) in IPF-fibroblasts. (**A,B**) *COL1A1* mRNA levels of pulmonary fibroblasts derived from either control or IPF patients cultured in attached 3D collagen-I gels (**A**, *n* = 3) or ~28 kPa 2D PAA gels (**B**, *n* = 5); control patients were the same than those used in Figure 2 and Figure 3, respectively (**C**) Potential synergistic interaction between TGF-β1 and matrix stiffening in terms of *COL1A1* in control and IPF-fibroblasts cultured in 3D collagen-I gels assessed as in Figure 4A. (**D**–**F**) Corresponding *MMP1* mRNA levels (**D**,**E**) and potential synergy between TGF-β1 and matrix stiffening (**F**) in control and IPF-fibroblasts cultured as in A-B and C, respectively. (**G**) Representative Western blot showing Akt^pS473^, total Akt and β-actin of control fibroblasts and IPF fibroblasts stimulated with TGF-β1 for 6 h; (right) corresponding densitometry analysis of Akt^pS473^/Akt in control (*n* = 4) and IPF fibroblasts (*n* = 4). Two group comparisons were performed by Student’s *t*-test.

Finally, we examined to what extent fibroblast responses to the mechanochemical pro-fibrotic conditions provided by stiff culture substrata and TGF-β1 become aberrant in IPF in terms of both *COL1A1*-*MMP1* expression and the activity of the FAK-Akt pathway. For this purpose, primary fibroblasts isolated from IPF patients were cultured in either pre-fibrotic 3D collagen-I gels or fibrotic 2D collagen-I coated PAA gels as described in Figure 1, and their responses to TGF-β1 were compared to those observed in fibroblasts from control pulmonary tissue. IPF-fibroblasts exhibited consistently larger mRNA *COL1A1* levels when cultured in stiff substrata in the presence of TGF-β1 compared to fibroblasts from control tissue, although to a larger extent in 3D gels (~1.7-fold) (Figure 6A) than 2D PAA gels (~1.2-fold) (Figure 6B). Likewise, *MMP1* mRNA levels were consistently larger in IPF-fibroblasts compared to normal fibroblasts in 3D cultures (~4.7-fold) (Figure 6D) and 2D PAA gels (~1.15-fold) (Figure 6E), although the latter difference did not attain statistical significance. These results reveal that IPF-fibroblasts exhibit abnormally high mRNA levels of *COL1A1* and possibly of *MMP1* when cultured in pro-fibrotic mechanochemical conditions. Furthermore, we analyzed the potential synergy between matrix stiffening and TGF-β1 in IPF-fibroblasts by computing Δ*R_stiff_*/Δ*R_soft_* using data gathered with the 3D collagen-I gel assay. Remarkably Δ*R_stiff_*/Δ*R_soft_* of *COL1A1* was ~2-fold higher in IPF-fibroblasts compared to control fibroblasts with statistical significance (Figure 6C), revealing an aberrant positive synergy between matrix stiffening and TGF-β1 in IPF-fibroblasts far beyond that of normal fibroblasts. In contrast, *MMP1* data elicited Δ*R_stiff_*/Δ*R_soft_* >1 in IPF-fibroblasts, whereas such ratio was negative in control fibroblasts (Figure 6F), revealing an aberrant positive synergy between matrix stiffening and TGF-β1 in IPF-fibroblasts in terms of *MMP1* expression.

With regards to the activity of the FAK/Akt pathway, we recently reported enhanced FAK^Y397^ in IPF-fibroblasts compared to fibroblasts from control tissue upon TGF-β1 stimulation using standard 2D cultures [40]. To assess the FAK/Akt pathway further, we examined Akt activity in IPF-fibroblasts and control fibroblasts cultured in 2D and activated by TGF-β1, and found a larger increase in Akt^pS473^ in IPF-fibroblasts after 6 h as shown in Figure 6G, although it did not attain statistical significance. Thus, these results suggest that the enhanced activity of the FAK/Akt pathway may underlie, at least in part, the aberrant collagen homeostasis in IPF-fibroblasts.

## 3. Discussion

### 3.1. First Evidence That the Inverse Regulation of COL1A1 and MMP1 mRNA Levels by TGF-β1 in Normal Fibroblasts Does Not Hold in Soft (Normal-Like) 3D Microenvironments

How the mechanical microenvironment and TGF-β1 cooperate to control collagen deposition in normal and fibrotic conditions had remained poorly understood. Of note, previous studies with 2D cultures had revealed that TGF-β1 transcriptionally increases *COL1A1* while decreasing *MMP1* in normal fibroblasts [14,15]. Such inverse regulation was also observed in the present study in fibroblasts from control tissue cultured in both pre-fibrotic and fibrotic-like rigidities elicited by attached 3D collagen-I gels and stiff 2D PAA gels, respectively. However, to our surprise the inverse regulation of *COL1A1* and *MMP1* mRNA levels by TGF-β1 was also observed in soft 2D PAA gels but not in soft (normal-like) 3D collagen-I gels, even though both gel types exhibited similar stiffnesses. This discrepancy reveals for the first time that the inverse regulation of *COL1A1* and *MMP1* mRNA levels by TGF-β1 is not universal but rather strongly dependent on the mechanics and dimensionality of the microenvironment.

Since 3D cultures are expected to be better tissue surrogates than 2D cultures [41,42], our observations in soft 3D collagen-I gels are likely to be more physiologically relevant than those in soft 2D PAA gels. Because an inverse transcriptional regulation is expected to contribute to collagen deposition by reducing collagen-I degradation while increasing its expression [6,7], it is conceivable that the loss of such inverse regulation in soft (normal-like) 3D microenvironments may prevent a large increase in collagen-I deposition, which could be beneficial for normal wound healing. However the functional implications of the loss of the inverse regulation of *COL1A1*-*MMP1* mRNA levels by TGF-β1 in soft 3D microenvironments remain to be elucidated.

### 3.2. First Direct Quantitative Evidence of Transcriptional Synergy between Matrix stiffening and TGF-β1 and the Potential Role of the FAK/Akt Pathway

Few previous studies have examined the impact of matrix stiffening alone on fibroblasts in terms of *COL1A1* transcription [7,17,18,43], and even less in terms of *MMP1* [7]. In the absence of TGF-β1, 2D PAA gels and 3D cultures elicited an increase of *COL1A1* in the range of ~10–25%, which is similar to the ~20% increase in *COL1A1* reported in cardiac and pulmonary fibroblasts cultured in 2D PAA gels [7,18]. However our observed increase in *COL1A1* was lower than the 150–190% increase reported in other studies using either 3D cultures or 2D PAA gels [17,18,43], which may be associated with distinct experimental conditions or tissue of origin. On the other hand, we found that matrix stiffening in the absence of TGF-β1 increased *MMP1* by ~1% in 3D cultures whereas it decreased *MMP1* by ~80% in 2D PAA gels, which is similar to the 70% decrease in 2D PAA gels reported elsewhere [7]. However to our knowledge this is the first study that provides direct quantitative evidence for an inverted transcriptional synergy between matrix stiffening and TGF-β1 in fibroblasts, which was positive for *COL1A1* and negative for *MMP1*.

With regards to mechanisms, four lines of evidence support that the FAK/Akt pathway may have an important role in the transcriptional co-regulation of collagen homeostasis by both TGF-β1 and matrix stiffening. First, Akt is a known downstream target of FAK activity through PI3k [37,44,45]. Second, the activities of FAK^Y397^ and Akt^S473^ are upregulated by both matrix rigidity [19,35] and TGF-β1 [38,46]. Third, pharmacologic inhibition of Akt or its downstream target mTOR has consistently elicited the downregulation of collagen-I concomitantly with the upregulation of MMP1 in fibroblasts [15,47,48]. Fourth, Akt was required for the increase of collagen expression by mechanical stretch elsewhere [49]. However, in addition to the FAK/Akt pathway, we can envision other potential mechanisms underlying the transcriptional regulation of collagen homeostasis by matrix rigidity and TGF-β1 based on previous observations, including direct changes in TGF-β expression induced by matrix stiffening [50,51], osteopontin upregulation [52] or a crosstalk between the matrix stiffening-sensitive transcription factors YAP/TAZ and regulatory SMADs [53]. Thus, the final elucidation of the key mechanism requires future investigations.

### 3.3. Comparing Fibroblast Responses to Matrix Stiffening and TGF-β1 Using 2D and 3D Culture Assays Provides New Insights on the Role of Matrix Dimensionality in Regulating Transcriptional Responses in Fibroblasts

3D cultures are expected to be more physiologically relevant than traditional 2D cultures because they provide critical cues from the tissue microenvironment, including adhesion to native ECM components and a 3D architecture [41,42]. However current 3D cultures based on collagen-I gels are somewhat limited for mechanobiology studies in the context of fibrosis because they do not capture the full stiffening reported in pulmonary fibrosis [7,24,54]. In contrast 2D PAA gels cover the stiffening of pulmonary fibrosis but miss the 3D architecture of tissues. Despite their differences, both 3D cultures and 2D PAA gels revealed consistently an upregulation of *COL1A1* concomitantly with a downregulation of *MMP1* upon TGF-β1 stimulation in the stiffest gel conditions but not in the softest conditions. This discrepancy revealed that TGF-β1 transcriptional regulation of *MMP1* depends strongly on the dimensionality of the fibroblast microenvironment. Likewise we found marked and opposite differences in the total levels of mRNA in *COL1A1* and *MMP1* between 3D cultures and 2D PAA gels, in which *COL1A1* were higher and *MMP1* lower in the latter gels. The larger mRNA *COL1A1* levels in 2D PAA gels are consistent with previous studies reporting increased metabolic and transcriptional activities in cells cultured in 2D compared to 3D gels [55,56], which was associated in part with an increased demand for endogenous ECM in the former conditions [56,57]. In contrast the larger *MMP1* expression in 3D cultures could reflect an excessive collagen-I concentration in these gels. On the other hand it is worth noting that a previous study reported cellular responses in response to matrix stiffening using both 2D PAA gels and 3D floating/attached gels that were consistent qualitatively but not quantitatively in terms of expression of differentiation markers [27]. Collectively the latter study and our results support that comparing 2D and 3D gels with tunable elasticity is a suitable approach to identify those cellular responses that are largely driven by matrix stiffening rather than matrix dimensionality.

### 3.4. Aberrant Collagen Homeostasis and FAK/Akt Activity in IPF-Fibroblasts, and How They May Contribute to Fibrosis Progression

The regulation of *COL1A1* mRNA by both matrix stiffening and TGF-β1 in IPF-fibroblasts analyzed in this study were aberrant in two complementary aspects. First, the total mRNA levels of *COL1A1* were higher in IPF-fibroblasts compared to control fibroblasts, which is consistent with previous observations [40,58]. Second, the positive interaction between matrix stiffening and TGF-β1 was also larger in IPF-fibroblasts than in fibroblasts from control tissue. Because there are numerous examples of positive correlations between changes in collagen-I at mRNA and protein levels in fibroblasts [7,15,59], it is expected that the two aberrant features of *COL1A1* mRNA regulation of IPF-fibroblasts reported here do contribute to the excessive collagen-I deposition at the protein level in IPF. However it should be borne in mind that collagen-I deposition is highly complex, for it is regulated at the transcriptional, posttranscriptional and secretory levels [4,5,60]. Therefore it is conceivable that the excessive amount of collagen-I in IPF is contributed by regulatory processes other than the increased mRNA reported here.

Of note our study also provided evidence that the aberrant *COL1A1* expression in IPF-fibroblasts may involve the FAK/Akt pathway, since both FAK^Y397^ and Akt^S473^ were upregulated in stiff substrata in response to TGF-β1 compared to control fibroblasts, and FAK deletion was sufficient to abrogate the increase in both Akt^S473^ and *COL1A1* upon TGF-β1 stimulation. In support to our interpretation, FAK^Y397^ activity was found to be increased within fibroblast foci in IPF [39,61,62]. Likewise both FAK and Akt activities were upregulated in fibrotic areas in the bleomycin model of pulmonary fibrosis [63]. Moreover, it has been recently reported that Akt controls the activity of LARP6 in lung fibroblasts, which is an essential regulator of both the translation of collagen mRNA and the synthesis of collagen-I [64]. All these observations may have important translational consequences, for they support that targeting the FAK/Akt pathway may be a suitable therapeutic approach against IPF progression. In line with this hypothesis, different protein kinase inhibitors have successfully reduced collagen deposition and myofibroblast population (two critical markers of organ fibrosis) concomitantly with a reduction in the phosphorylation/activation of FAK and Akt in the bleomycin model of pulmonary fibrosis [39,63,65].

The response of IPF-fibroblasts to pro-fibrotic biochemical and mechanical stimuli was also aberrant in terms of *MMP1* expression, since they did not exhibit a negative regulation unlike control fibroblasts. However it should be pointed out that, even though *MMP1* is among the molecules more significantly overexpressed in IPF compared with control lungs [8,66], the overall contribution of fibroblasts to the total *MMP1* levels in IPF remains uncertain. Thus, previous studies have reported that *MMP1* expression in IPF is largely associated with epithelial cells, whereas it is virtually absent in fibroblast foci [67,68]. Likewise the role of MMP1 in IPF remains unclear [8], although it has been speculated to contribute to epithelial migration [1]. Unlike *MMP1*, *MMP2* upregulation in IPF has been associated, at least in part, with IPF-fibroblasts, and has been implicated in the tissue migration of fibrocytes, particularly through basement membrane proteins, thereby potentially facilitating the accumulation of these cells in IPF [8,69].

In summary our work provides new insights on the mechanobiology of normal collagen homeostasis in fibroblasts and how it becomes awry in IPF, and support an important role of the FAK/Akt pathway in collagen homeostasis in both normal and fibrotic conditions.

## 4. Materials and Methods

### 4.1. Isolation of Patient-Derived Lung Fibroblasts

All primary fibroblasts were isolated by outgrowth of tissue explants from pulmonary tissue as reported elsewhere [70], and frozen stored in liquid nitrogen at passage 2–3 until use. All protocols were approved by the Ethics Committees of the *Hospital Clínic de Barcelona* and the *Universitat de Barcelona*, and patients gave their informed consent. Samples from histologically normal pulmonary tissue were obtained from either patients undergoing surgical pleurodesis to treat recurrent spontaneous pneumothorax (*n* = 3) [40,70] or from uninvolved pulmonary tissue of surgical patients with non-small cell lung cancer (*n* = 6) [35], and were used to obtain primary control fibroblasts. Tissue samples from IPF patients were obtained from lung biopsies of patients exhibiting the histopathological pattern of usual interstitial pneumonia (*n* = 5).

### 4.2. Cell Culture in Standard 2D Tissue Culture Plastic (2D Culture)

The normal human fibroblast cell line CCD-19Lu (ATCC) and primary fibroblasts were propagated in standard 2D cultures as previously described [35,40]. In brief, fibroblasts were fast thawed and maintained in fibroblast culture media containing DMEM supplemented with 10% FBS (FBS Gold, PAA Laboratories, GE Healthcare, Little Chalfont, UK) and antibiotics. Primary fibroblasts were used up to 5–6 passages to prevent replicative senescence. Mouse embryonic fibroblasts (MEFs) from wild-type (FAK^+/+^) or FAK-deficient (FAK^−/−^) mice (ATCC) were cultured in DMEM media as reported elsewhere [39]. All cultures were kept in a 5% CO_2_ humidified incubator at 37 °C. For 2D culture experiments, MEFs were seeded as either 12 × 10^3^ cells/cm^2^ (FAK^+/+^) or 20 × 10^3^ cells/cm^2^ (FAK^−/−^) in serum-free culture medium with or without 5 ng/mL human TGF-β1 (R&D Systems, Minneapolis, MN, USA) for ~4 days.

### 4.3. Cell Culture in 3D Collagen-I Gels

Collagen-I solution for 3D cultures was freshly prepared the day of the experiment as previously described [29,34]. In brief, acid soluble collagen-I (Cellagen IAC-50, Koken, Tokyo, Japan) was neutralized in DMEM to obtain a 4 mg/mL collagen solution. All 3D cultures were conducted in 24 well plates. First, wells were pre-coated with a thin layer of collagen-I solution to prevent cell migration. Second, fibroblasts were trypsinized and resuspended with collagen-I solution at 0.9 × 10^6^ cells/mL, and 215 μL of this mixture were added to a well, incubated at 37 °C for 30 min, and immediately hydrated with 90 μL of serum-free culture medium. All samples were kept in the incubator until use. After 24 h, half of the gels were gently detached from the well container edge with a sterile spatula, whereas the other half remained attached [27], and culture medium was replaced by fresh serum-free medium in the absence or presence of 5 ng/mL human TGF-β1 (R&D Systems) for ~4 days.

### 4.4. Cell Culture in 2D PAA Gels

Collagen-I–coated polyacrylamide (PAA) gels were obtained following a similar protocol described elsewhere [27,35,71]. In brief, gels were prepared by mixing variable ratios of acrylamide (AA; 3–12%) and bis-acrylamide (BIS; 0.22–0.6%) (Bio-Rad, Hercules, CA, USA). After polymerization, the PAA gel surface was derivatized with Sulfo-SANPAH (Thermo Scientific, Waltham, MA, USA) and coated with 0.1 mg/mL collagen-I (Millipore, Billerica, MA, USA). Fibroblasts were seeded at 8 × 10^3^ cells/cm^2^ in serum-free culture medium with or without 5 ng/mL human TGF-β1 (R&D Systems) for ~4 days.

### 4.5. Elasticity Nanoindentation Measurements by AFM

The Young’s elastic modulus *E* of 3D collagen-I gels and 2D PAA gels was assessed with a home-made stand-alone AFM adapted to an inverted optical microscope as described in detail elsewhere [27,36]. In brief, AFM nanoindentation measurements were performed with low spring constant cantilevers with pyramidal tips (nominal *k* = 0.03 N/m) (Microlever, Veeco, Santa Barbara, CA, USA), which were calibrated using the thermal noise method [36]. For each gel location, three force-displacement curves (*F-z* curves) were acquired at a moderate loading force (~1 nN); *E* of each *F-z* curve was computed by least-squares fitting of a contact elastic model, and averaged over the three *F-z* curves [27]. The same protocol was applied on at least 9 random gel locations to elicit the final *E* estimate of the hydrogel.

### 4.6. qRT-PCR

Cell cultures were performed in triplicates for qRT-PCR analysis, which was conducted as reported elsewhere [40]. Briefly for each sample, total RNA was isolated using the RNeasy Mini kit (QIAGEN) and reverse-transcribed into cDNA using the High Capacity cDNA Reverse Transcription Kit and RNase inhibitor (Applied Biosystems, Foster City, CA, USA). Unless otherwise indicated, real-time PCR reactions were performed on 20–50 ng of each cDNA sample using TaqMan gene-specific primer pairs and probes for human genes encoding *COL1A1* (Hs00164004_m1), *MMP1* (Hs00233958_m1), *MMP2* (Hs01548724_m1) and *POLR2A* (Hs00172187_m1, used as a reference gene) and for mouse genes encoding for *Col1a1* (Mm00801666_g1), *Mmp1a* (Mm00473485_m1) and *Polr2a* (Mm00839502_m1, used as a reference gene) (Applied Biosystems). Reactions were carried out for 40 cycles (95 °C for 21 s and 60 °C for 20 s) in a 7900HT Fast Real-Time PCR System (Applied Biosystems). Relative gene expression with respect to *POLR2A* (for human) or *Polr2a* (for mouse fibroblasts) was assessed as *k*·2^−Δ*Ct*^, where *k* is the ratio of the threshold numbers used for the target and the endogenous genes, respectively, and Δ*C*t is the difference between the average *C*t of the target and the reference (endogenous) genes [72].

### 4.7. Assay of MMP-2 Activity

The activity of MMP-2 was visualized by gelatin zymography as described elsewhere [73]. Briefly, protease inhibitors were added to each cell culture (complete EDTA-free protease inhibitor cocktail, Roche, Basel, Switzerland), and the corresponding conditioned media was collected, concentrated (Ultrafiltration Amicon, Milipore, Burlington, MA, USA) and subjected to electrophoresis in 10% gelatin gels (Invitrogen, Carlsbad, CA, USA). Afterwards, proteins were renatured with a renaturing buffer (Invitrogen) and gelatinase activity was observed upon incubation with a developing buffer (Invitrogen) for 18 h at 37 °C. As a negative control, 10 nM EDTA was added to the developing buffer. Gelatin gels were stained with Coomassie blue. Regions of enzymatic activity (proteolysis) appeared as clear bands against a dark background.

### 4.8. Western Blotting

FAK and Akt activities were assessed by Western blotting as reported elsewhere [35]. In brief, fibroblasts were lysed in extraction buffer supplemented with phosphatase and protease inhibitors. Equal protein amounts were separated on a 10% Mini-PROTEAN TGX precast gels (Bio-Rad), transferred to a PVDF membrane (GE Healthcare, Marlborough, MA, USA), blocked and incubated overnight with primary antibodies against Akt, Akt^pS473^ (9272 and 4060; Cell Signaling Technology, Danvers, MA, USA), and α-tubulin (2144, Cell Signaling) or anti-β-actin (Sigma, Saint Louis, MI, USA). The latter two proteins were used as loading controls. Protein bands were labeled and visualized by chemiluminescence (ImageQuant LAS 4000, GE Healthcare), and the corresponding band intensities were quantified with Image J.

### 4.9. Statistical Analysis

Comparisons of results obtained from fibroblasts from the same patients were carried out using paired Student’s *t*-test. Otherwise we used unpaired Student’s *t*-test. Statistical analyses were performed with SigmaStat (Systat Software, San Jose, CA, USA). Statistical significance was assumed at *p* < 0.05. Data are given as mean ± SE.

## Figures and Tables

**Table 1:** Reported Young’s elastic moduli or elastance (*) of normal pulmonary tissue.

Species	Young’s Modulus or Elastance (*)	Bulk/Nano-Scale (Technique)	Reference
human	3–3.6 kPa	bulk (indentation test)	[23]
porcine	~10 kPa	bulk (magnetic resonance elastography)	[22]
porcine	≤4 kPa	bulk (magnetic resonance elastography)	[32]
dog	~5 kPa	bulk (uniaxial oscillatory stretch)	[33]
mouse	0.1–5 kPa (*)	nanometer-scale (AFM)	[7]
rat	5–10 kPa (*)	bulk (uniaxial oscillatory stretch)	[24]
rat	13 kPa	bulk (uniaxial oscillatory stretch)	[26]

## Supplementary Materials

The following are available online at www.mdpi.com/1422-0067/18/11/2431/s1.

## Abbreviations

AFM	atomic force microscopy
α-SMA	alpha smooth muscle actin
*E*	Young’s elastic modulus
FAK	focal adhesion kinase
TGF-β1	transforming growth factor beta1
