# Supplementary material for: Dysregulated Collagen Homeostasis by Matrix Stiffening and TGF-β1 in Fibroblasts from Idiopathic Pulmonary Fibrosis Patients: Role of FAK/Akt
Source: Int J Mol Sci. 2017 Nov 16;18(11):2431. doi: 10.3390/ijms18112431 (PMC5713399; doi:10.3390/ijms18112431)
Supplement: Supplementary file 1 [file ijms-18-02431-s001.pdf]

SUPPLEMENTARY FIGURE S1: potential synergy between matrix stiffness and TGF-b1

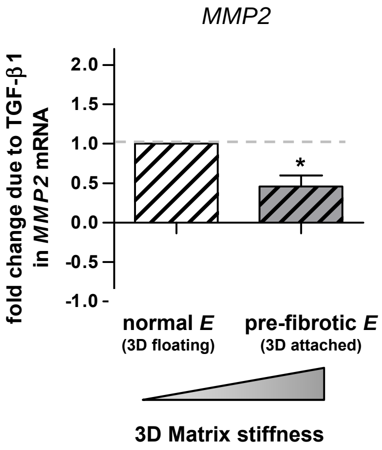

Supplementary Figure S1. Difference in *MMP2* expression with our without TGF-β1 measured in soft and stiff substrata (shown in Figure 2) normalized to that obtained in soft substrata using 3D collagen-I gels in pulmonary fibroblasts derived from healthy donors (n=3). Further details are given in the main text. * $P \leq 0.05$, with respect to no change (1) was determined by Student's t-test.

SUPPLEMENTARY FIGURE S2: Time-course of Akt activation by TGF-β1

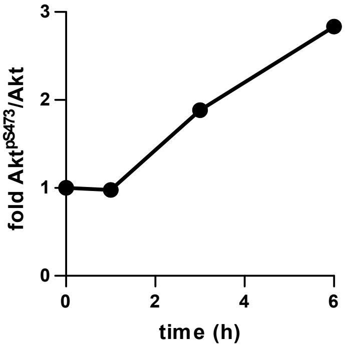

Supplementary Figure S2. Quantification of the Western-blot shown in Fig. 5A corresponding to the activity of Akt induced by TGF-β1 as a function of time in control fibroblasts cultured in 2D.
